# Supplementary material for: Acss2 Deletion Reveals Functional Versatility via Tissue-Specific Roles in Transcriptional Regulation
Source: Int J Mol Sci. 2023 Feb 12;24(4):3673. doi: 10.3390/ijms24043673 (PMC9964712; doi:10.3390/ijms24043673)
Supplement: Supplementary file 1 [file ijms-24-03673-s001.zip › ijms-2130167-supplementary.pdf]

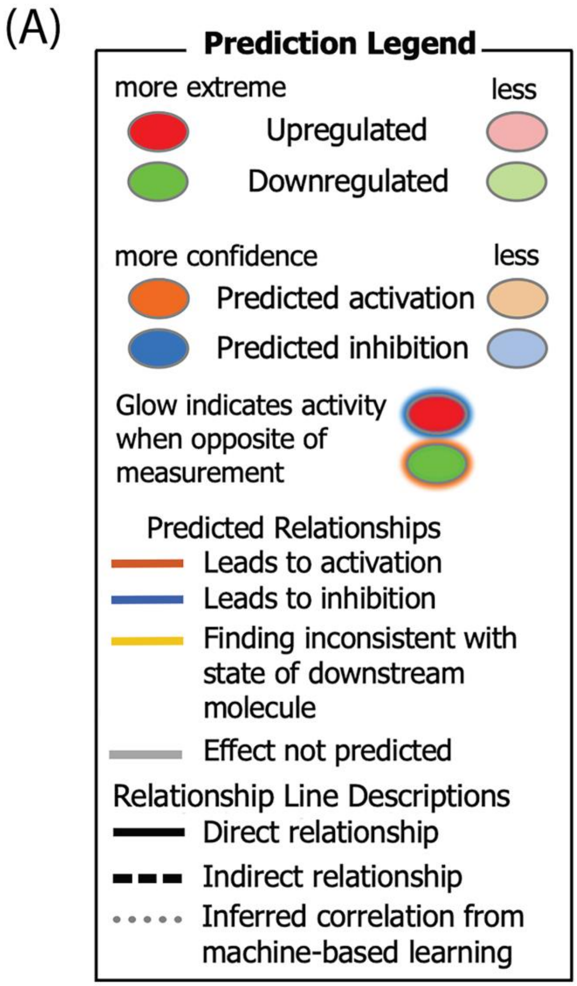

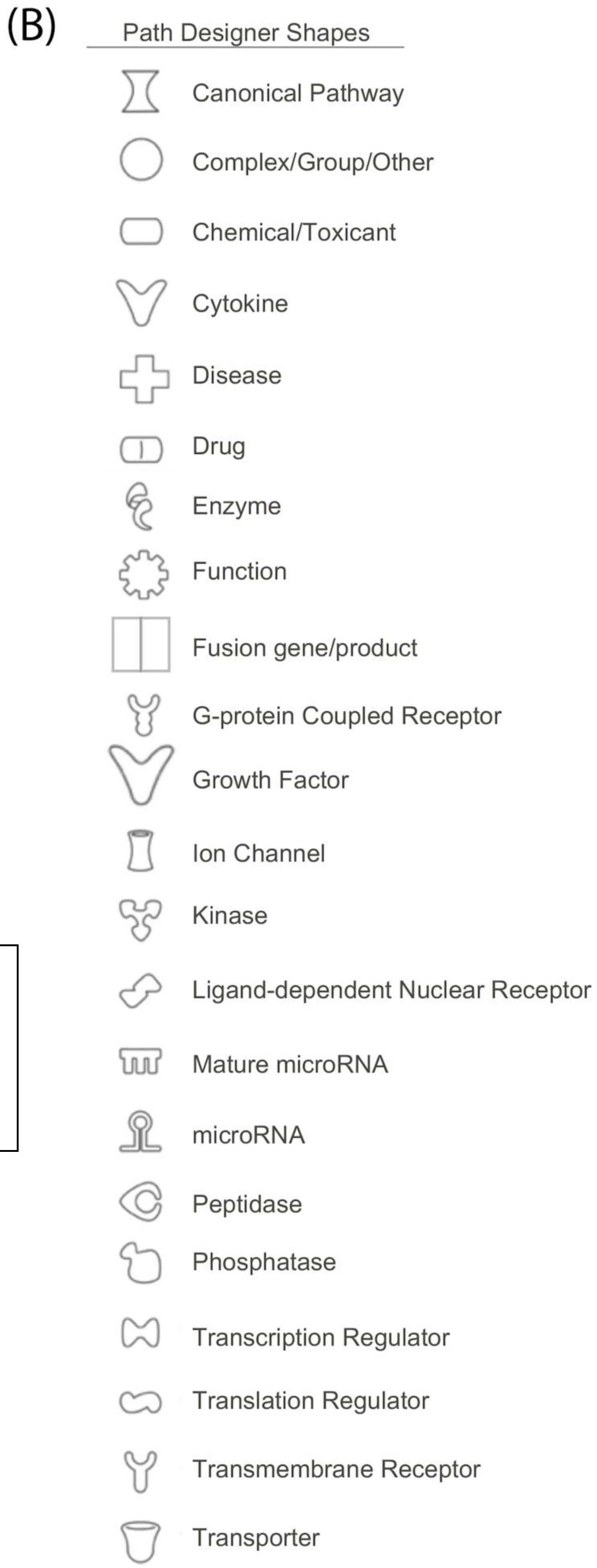

Supplementary Figure S1: (A) The color scheme indicates the value of the differential gene expressions. **(B)** Shapes indicate the type and function of the DEG products.

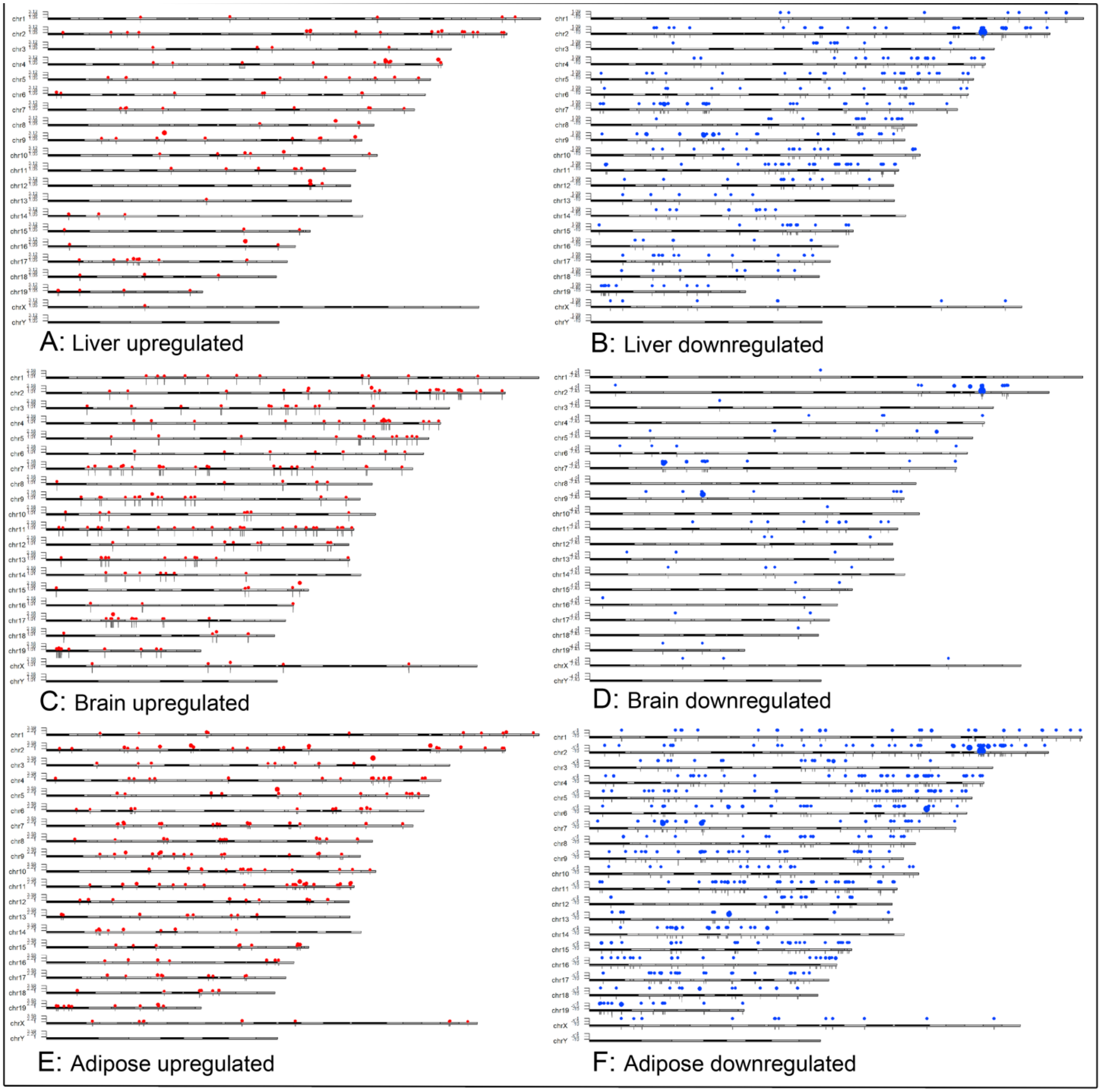

Supplemental Figure S2: Karyoplot of DEGs in the 3 organ systems: (A) Liver upregulated gene distribution; (B) Liver downregulated gene distribution; (C) Brain upregulated gene distribution; (D) Brain downregulated gene distribution; (E) Adipose upregulated gene distribution; (F) Adipose downregulated gene distribution. The figure indicates the geographical location of the perturbed genes on each chromosome. The X-axis indicates the chromosomal location. The size of the dot on the Y-axis corresponds to the log fold change of the gene determined experimentally. The general pattern across the 3 organ systems shows the gene expression perturbation to be relatively organ-specific and widespread across all chromosomes with the exception of the Y-chromosome.

<u>Supplementary Table S1:</u> Fatty acid concentration data (µg/ml)

Sample Name	Adipose-186	Adipose-187	Adipose-189	Adipose-190	Adipose-191	Adipose-194	Adipose-196	Adipose-197	Adipose-198	Adipose-199
**Dodecanoic acid**	2.62	2.19	2.77	2.75	3.41		2.50	3.49		2.94
**Myristic acid**	21.02	17.52	24.19	25.38	22.94	13.52	13.67	16.14	11.46	22.82
**Pentadecanoic acid**	85.48		117.26	106.53	82.58	60.61	66.39	81.87	53.58	95.04
**Palmitoleic acid**	3.06		3.77	2.46	2.37	2.33	1.75	2.74		2.84
**Palmitic acid**	1149.12	1346.09	1246.80	1125.24	1067.61	1110.33	1120.78	1218.40	963.45	1553.07
**Heptadecenoic acid**		8.26								
**Heptadecanoic acid**	1.27	1.47	1.33	1.41	1.14	0.76	0.92	1.02	0.78	1.99
**Octadecatetraenoic acid (Stearidonic acid)**										
**a-Linolenic acid**	5.56	7.23	4.95	4.41	5.92	6.19		5.24	4.83	5.90
**g-Linolenic acid**	22.95	18.84	27.95	24.71	31.77	38.24		23.66		28.52
**Linoleic acid**	125.91	112.04	124.16	101.52	93.51	142.13	122.78	107.96	102.09	112.49
**Oleic acid**	50.90	44.99	50.20	36.86	34.98	48.13	37.17	43.75	43.43	54.11
**Stearic acid**	148.16	168.54	156.47	140.80	95.95	128.80	139.82	150.48	107.05	289.85
**Eicosapentaenoic acid**	1.17	1.68	1.36	1.39	1.31	1.58	1.51	1.16	1.25	0.84
**Arachidonic acid**	8.52	8.69	9.66	7.49	2.47	12.03	10.26	7.59	3.33	7.79
**Mead acid**										
**Dihomo-g-linolenic acid**										
**Eicosadienoic acid**	2.08		1.93				2.47			
**Eicosenoic acid**	2.78	2.54	3.00		2.10	2.51	1.64	2.04	1.93	2.86
**Arachidic acid**										0.41
**Docosahexaenoic acid**	8.90		13.98	9.91	5.40	17.08	15.55	12.13		11.04
**Docosapentaenoic acid (n-3)**										
**Docosapentaenoic acid (n-6)**										
**Docosatetraenoic acid**										
**Docosatrienoic acid**										
**Docosadienoic acid**										
**Docosenoic acid**										
**Docosanoic acid (Behenic acid)**										
**Nervonic acid**										
**Lignoceric acid**	7.99	9.97	9.18	8.16	8.73	8.29	8.47	9.27	8.88	9.13
**Hexacosanoic acid**	0.94	1.13	0.93	0.92	0.95	0.92	0.94	1.02	1.04	1.03
	**Brain-186**	**Brain-187**	**Brain-189**	**Brain-190**	**Brain-191**	**Brain-194**	**Brain-196**	**Brain-197**	**Brain-198**	**Brain-199**
**Dodecanoic acid**	3.60	1.72	4.12	2.95	2.42	2.49	3.58	3.82	3.37	3.16
**Myristic acid**	34.14	24.10	33.77	28.09	31.13	26.66	22.00	24.64	35.33	31.87
**Pentadecanoic acid**	130.80	133.43	201.83	138.26	185.88	121.63	102.25	119.98	267.43	228.04
**Palmitoleic acid**	6.83	10.17	13.31	10.89	12.57	9.68	8.77	9.68	15.71	16.76
**Palmitic acid**	10362.41	10609.92	13405.59	10059.27	25017.87	7805.85	10490.33	11065.71	8151.77	14155.73
**Heptadecenoic acid**	9.17	15.52	22.76	17.21	21.66	14.60	11.12	13.32	29.86	27.96
**Heptadecanoic acid**	1.01	0.86	1.07	0.76	1.12	0.86	0.62	0.64	1.24	0.88
**Octadecatetraenoic acid (Stearidonic acid)**										
**a-Linolenic acid**			5.77		2.54				3.20	
**g-Linolenic acid**			15.03	27.54						
**Linoleic acid**	59.64	69.89	88.81	84.61	66.58	84.68	69.73	58.74	78.44	94.74
**Oleic acid**	933.97	974.15	1145.16	1189.27	1178.10	1438.32	1388.44	1163.57	1184.71	1828.51
**Stearic acid**	833.04	728.55	758.29	779.90	767.63	871.40	931.19	944.04	893.59	778.44
**Eicosapentaenoic acid**	0.65	1.09	0.93	1.06	0.79	0.99	0.83	0.75	0.89	0.93
**Arachidonic acid**	237.74	268.86	330.94	320.85	275.15	372.00	316.54	312.04	338.42	423.89
**Mead acid**	2.29	2.72	3.43	3.24	2.81	3.25	2.81	2.32	2.95	3.05
**Dihomo-g-linolenic acid**	1.03	1.25			1.16	1.47	1.22	1.14	1.55	1.58
**Eicosadienoic acid**	23.10	25.96	39.30	35.66	25.22	42.41	35.40	21.77	29.03	31.64
**Eicosenoic acid**	146.12	141.85	240.04	215.89	170.36	275.21	274.22	165.91	167.89	215.64
**Arachidic acid**	1.05	1.03	1.48	1.38	1.09	1.67	1.51	1.11	1.29	1.34
**Docosahexaenoic acid**	1319.87	1476.67	1657.11	1696.73	1503.57	1775.30	1472.68	2331.50	1654.62	15037.17
**Docosapentaenoic acid (n-3)**	46.59	55.10	65.18	56.37	59.11	70.03	59.73	54.44	69.97	78.54
**Docosapentaenoic acid (n-6)**										
**Docosatetraenoic acid**	92.40	93.47	136.42	127.73	106.41	145.23	121.82	113.53	130.17	133.66
**Docosatrienoic acid**	10.29	9.47	14.84	13.06	10.96	15.27	13.12	11.87	14.19	13.57
**Docosadienoic acid**	8.24	9.05	16.76	15.34	11.67	17.44	14.48	9.27	16.68	14.10
**Docosenoic acid**	27.30	26.74	44.69	38.15	32.59	48.87	48.94	32.67	34.16	40.11
**Docosanoic acid (Behenic acid)**	2.66	2.79	4.90	3.76	3.22	4.51	4.49	3.33	3.24	3.76
**Nervonic acid**	14.29	14.22	27.31	21.35	19.68	30.14	29.35	16.94	18.46	21.21
**Lignoceric acid**	18.32	19.07	28.71	23.72	23.35	29.63	30.58	20.82	24.80	25.41
**Hexacosanoic acid**	0.98	1.15	1.28	1.09	1.21	1.27	1.23	1.14	1.47	1.29
	**Liver-186**	**Liver-187**	**Liver-189**	**Liver-190**	**Liver-191**	**Liver-194**	**Liver-196**	**Liver-197**	**Liver-198**	**Liver-199**
**Dodecanoic acid**	3.39	4.90		3.14	3.41	3.59		3.39		3.11
**Myristic acid**	14.24	18.24	17.82	17.61	15.11	30.77	13.94	14.74	29.93	17.03
**Pentadecanoic acid**	80.11	112.38	117.36	107.24	78.51	158.69	87.51	86.55	148.31	93.17
**Palmitoleic acid**	4.40	5.29	7.87	8.63	9.99	5.72	6.73	6.17	6.60	5.46
**Palmitic acid**	7787.85	8533.11	6911.46	6297.65	9688.65	8005.33	9877.54	8741.30	8784.47	8529.28
**Heptadecenoic acid**		8.31	9.58	10.18	7.45	14.09	9.88	9.26	9.06	
**Heptadecanoic acid**	0.89	1.19		1.20	0.92	1.76	0.92		1.98	0.81
**Octadecatetraenoic acid (Stearidonic acid)**										
**a-Linolenic acid**	35.46	39.51	42.96	35.68	39.90	43.99	34.37	39.07	34.68	37.91
**g-Linolenic acid**	179.50	216.44	236.39	194.03	202.07	226.55	180.83	213.68	177.09	199.74
**Linoleic acid**	853.49	1163.69	1279.84	946.86	1078.11	1093.06	1150.13	1268.21	906.75	1071.78
**Oleic acid**	164.68	184.13	260.17	265.71	214.68	181.01	226.08	221.46	188.75	176.08
**Stearic acid**	577.16	589.62	657.63	666.80	510.53	641.88	573.92	628.41	678.75	553.44
**Eicosapentaenoic acid**	1.74	2.45	1.81	1.53	2.11	1.98	1.80	2.12	1.56	2.20
**Arachidonic acid**	253.57	397.36	354.83	307.87	298.71	371.10	393.98	449.28	306.36	381.30
**Mead acid**	4.77	7.74	8.04	6.32	6.83	5.66	6.47	7.07	4.32	5.67
**Dihomo-g-linolenic acid**										
**Eicosadienoic acid**	56.29	71.76	62.95	59.13	33.52	60.58	62.28	82.27	46.60	66.16
**Eicosenoic acid**	14.04	15.70	19.41	22.00	13.49	14.90	18.28	19.39	14.31	15.43
**Arachidic acid**	1.50	1.39	1.68	1.78	1.38	1.52	1.10	1.31	1.30	1.31
**Docosahexaenoic acid**	671.77	974.13	959.57	772.88	723.00	982.30	1041.17	1034.07	753.62	923.76
**Docosapentaenoic acid (n-3)**	19.63	26.63	26.81	20.56		27.27	27.13	30.36	20.61	28.03
**Docosapentaenoic acid (n-6)**	20.84	27.41	46.20	28.93	55.50	31.14	36.03	37.04	22.71	28.38
**Docosatetraenoic acid**	5.01	7.71	8.21	7.13	7.45	6.87	9.63	8.66	6.29	7.51
**Docosatrienoic acid**								1.28		
**Docosadienoic acid**				4.05	3.00		3.06		4.21	
**Docosenoic acid**	2.62	2.66	3.17	4.21	3.08	3.08	2.96	3.01	2.60	3.05
**Docosanoic acid (Behenic acid)**			0.82	1.01	0.84					
**Nervonic acid**										
**Lignoceric acid**	9.02	9.80	8.96	10.62	9.97	10.44	8.59	8.39	9.76	8.74
**Hexacosanoic acid**	1.00	1.05	0.95	1.10	1.05	1.18	1.04	0.94	0.97	1.06

Supplementary Table S2.

Supplementary Table S2A - Liver. The -log (p-value) and z-score of the top 10 statistically significant (-log (p-value) > 1.3) and differentially regulated canonical pathways in liver tissue of *Acss2*$^{-/-}$ compared to wild-type mice.

Canonical Pathways	-log(p-value)	z-score
eNOS Signaling	2.91	0.816
Aldosterone Signaling in Epithelial Cells	2.87	-1
Ferroptosis Signaling Pathway	2.49	0.707
LXR/RXR Activation	2.44	1.633
Nitric Oxide Signaling in the Cardiovascular System	1.82	-0.447
NER (Nucleotide Excision Repair, Enhanced Pathway)	1.64	-0.447
Kinetochore Metaphase Signaling Pathway	1.58	-0.447
Xenobiotic Metabolism PXR Signaling Pathway	1.55	1.134
Role of MAPK Signaling in Promoting the Pathogenesis of Influenza	1.51	-2
AMPK Signaling	1.36	0.447

Supplementary Table S2B - Brain. The -log (p-value) and z-score of the top 10 statistically significant (-log (p-value) > 1.3) and differentially regulated canonical pathways in brain tissue of *ACSS2*$^{-/-}$ compared to wild-type mice.

Canonical Pathways	-log(p-value)	z-score
Sirtuin Signaling Pathway	4.95	1.414
Oxidative Phosphorylation	4.33	-2.828
3-phosphoinositide Biosynthesis	2.62	2.646
PI3K/AKT Signaling	2.2	-2.646
Superpathway of Inositol Phosphate Compounds	2.18	2.646
BEX2 Signaling Pathway	1.93	-2
Cyclins and Cell Cycle Regulation	1.91	-2

Fcγ Receptor-mediated Phagocytosis in Macrophages and Monocytes	1.66	2
Neuropathic Pain Signaling In Dorsal Horn Neurons	1.59	2
D-myo-inositol (1,4,5,6)-Tetrakisphosphate Biosynthesis	1.31	2

Supplementary Table S2C - Adipose. The -log (p-value) and z-score of the top 10 statistically significant (-log (p-value) > 1.3) and differentially regulated canonical pathways in adipose tissue of *Acss2*$^{-/-}$ compared to wild-type mice.

**Canonical Pathways**	**-log(p-value)**	**z-score**
Sirtuin Signaling Pathway	4.61	-2.828
Oxidative Phosphorylation	3.39	3.162
Glycolysis I	3.03	2.449
Toll-like Receptor Signaling	2.91	-1.633
iNOS Signaling	2.68	-2.236
Estrogen Biosynthesis	2.43	-1.633
LPS/IL-1 Mediated Inhibition of RXR Function	2.36	-2
Bupropion Degradation	2.26	-2
Spliceosomal Cycle	1.87	-2.236
Regulation Of The Epithelial Mesenchymal Transition By Growth Factors Pathway	1.84	-1.897
Acetone Degradation I (to Methylglyoxal)	1.44	-2

Supplementary Table S3.

Supplementary Table S3A - Liver. Expression of genes associated with disease and cellular functions, activation of "anemia" and "organ degeneration" in liver tissue of *Acss2*$^{-/-}$ compared to wild-type mice.

Symbol	Expr Log Ratio
C9	1.14
GBA	-1.16
GPX4	-1.16
MFGE8	-1.17
PIGA	-1.12
PITPNM2	-1.14
POLR3H	-1.09
REEP6	1.2
SLC25A38	-1.19
ZFP36L1	-1.47

Supplementary Table S3B - Liver. Expression of genes associated with disease and cellular functions, activation of "growth failure" in liver tissue of *Acss2*$^{-/-}$ compared to wild-type mice.

Symbol	Expr Log Ratio
BNIP3L	-1.21
FLT1	-1.12
GCK	-1.89
ITPR1	-1.2
SHMT2	1.12
TFRC	-1.45

Supplementary Table S3C - Liver. Expression of genes associated with disease and cellular functions, activation of “organismal death” in liver tissue of *Acss2*$^{-/-}$ compared to wild-type mice.

Symbol	Expr Log Ratio
BAG1	-1.22
BNIP3L	-1.21
FDXR	-1.08
HSD17B7	-1.34
ITPR1	-1.2
PRKACA	1.1
Rpl29 (includes others)	-1.54
STIP1	-1.21
TBX3	1.17

Supplementary Table S3D – Liver: Expression of genes associated with disease and cellular functions, inhibition of “infection by RNA virus” in liver tissue of *Acss2*$^{-/-}$ compared to wild-type mice.

Symbol	Expr Log Ratio
ATG5	1.09
ATMIN	-1.09
FGD6	-1.16
SAMD9L	1.41
WASF1	-1.11

Supplementary Table S4.

Supplementary Table S4A – Liver: Expression of genes associated with **Network 1**: “amino acid metabolism, carbohydrate metabolism, small molecule biochemistry”, in liver tissue of *Acss2*$^{-/-}$ compared to wild-type mice.

Symbol	Expr Log Ratio
ANKRD54	-1.08
Apol9a/Apol9b	1.24
ATP1B3	-1.13
AVEN	-1.1
BICD2	-1.14
COMMD9	-1.09
DMWD	-1.12
DYNLRB1	-1.32
EGLN3	-1.09
EIF6	-1.18
FADS2	-1.22
FOXRED1	-1.12
GALK1	-1.33
GLDC	1.16
GPN2	-1.59
IFT52	-1.3
JOSD2	-1.24
KLHDC10	-1.22
MSH6	-1.11
NDUFA8	1.1
NUCB1	-1.15
NUDT5	1.11

PALS2	1.13
PPAT	-1.17
SCAND1	1.61
SDE2	-1.19
SEL1L	-1.11
SHMT2	1.12
TCF25	-1.23
TKT	1.07
TRIP6	-1.17
UBE2Q1	-1.11
USH2A	-1.53

Supplementary Table S4B – Liver: Expression of genes associated with **Network 2**: “cellular development, cellular growth and proliferation, connective tissue development and function”, in liver tissue of *Acss2*$^{-/-}$ compared to wild-type mice.

Symbol	Expr Log Ratio
ARL1	-1.15
ASPSCR1	-1.22
BRMS1	-1.12
CDK5RAP3	-1.17
DAP3	1.13
DDX1	-1.15
Fus	1.19
G3BP2	1.18
GRB7	-1.31
HABP4	-1.09
HPN	1.22

ILVBL	-1.2
ING4	-1.11
LZTR1	-1.09
MRPS7	-1.31
NUAK2	-1.31
OLIG1	1.16
PLEKHG3	-1.12
POLD2	-1.1
PPP6C	-1.22
PXMP2	1.25
RBM42	-1.12
RIOK3	-1.34
SRPRA	-1.54
TAX1BP3	-1.19
TIMM10	-1.15
TMED4	-1.14
TRIM8	-1.15
TSPAN33	-1.13
UBXN6	-1.18
WFDC2	-2.02
YOD1	1.14

Supplementary Table S5.

Supplementary Table S5A – Brain: Expression of genes associated with disease and cellular functions, activated “cell viability of tumor cell lines” and “size of body” in brain tissue of *Acss2*$^{-/-}$ compared to wild-type mice.

Symbol	Expr Log Ratio
ARRB1	1.11
DLX1	1.09
IGFBP4	1.24
KCNC1	1.1
KLF6	1.16
LMNA	1.13
PER2	-1.15
RAB3A	1.01
RAB3D	1.06
THRSP	1.09

Supplementary Table S5B – Brain: Expression of genes associated with disease and cellular functions, activation of “DNA endogenous promoter” in brain tissue of *Acss2*$^{-/-}$ compared to wild-type mice.

Symbol	Expr Log Ratio
CCND1	-1.3
DEK	1.12
DLX1	1.09
LDB2	1.22
NDN	-1.1

Supplementary Table S5C – Brain: Expression of genes associated with disease and cellular functions, activation of “cell viability” and “organization of cytoskeleton” in brain tissue of *Acss2*$^{-/-}$ compared to wild-type mice.

Symbol	Expr Log Ratio
APP	-1.05
ARRB1	1.11
CCND1	-1.3
DCX	1.12
DUSP1	2.01
GNAO1	1.23
HSPB6	1.36
KLC1	1.15
NTRK2	1.19
PER2	-1.15
PIK3R2	1.05
POLR1D	1.1
RAB3A	1.01
RPS6KL1	1.12
SORL1	1.74
TRAF3	-1.23
WTIP	1.06

Supplementary Table S5D – Brain: Expression of genes associated with disease and cellular functions, "inhibition of organismal death" in brain tissue of *Acss2*$^{-/-}$ compared to wild-type mice.

**Symbol**	**Expr Log Ratio**
DLX1	1.09
KCNC1	1.1
NTRK2	1.19
OTX1	1.09

Supplementary Table S6.

Supplementary Table S6A – Brain: Expression of genes associated with **Network 1**: "connective tissue disorders, developmental disorder, hereditary disorder", in brain tissue of *Acss2*$^{-/-}$ compared to wild-type mice.

**Symbol**	**Expr Log Ratio**
ACTR3	1.17
ACTR3B	1.07
AIP	1.15
CSTF1	1.22
DCPS	1.11
DEK	1.12
EIF1B	-1.06
FRMD6	1.26
GALT	1.22
HBS1L	1.08
HSPB6	1.36
IDH3B	-1.05
KCNMA1	1.2
KEAP1	1.1
KLF6	1.16
NOSIP	-1.24
NUDC	1.16
NXT2	-1.06
PIH1D1	1.09
POLL	1.06
POLR1C	1.11
POLR1D	1.1

PRDX1	-1.04
SRPK1	1.15
STK4	1.35
TRIP12	1.11
UBE3A	1.1
UBTD2	1.15

Supplementary Table S6B – Brain: Expression of genes associated with **Network 2:** "metabolic disease, neurological disease, organismal injury and abnormalities", in brain tissue of *Acss2*$^{-/-}$ compared to wild-type mice.

**Symbol**	**Expr Log Ratio**
ATP5F1C	-1.05
ATP6V0A2	1.1
Cd59a	1.61
COX10	-1.07
CTDSPL	1.09
ELMO2	-1.23
GHITM	1.15
GLRX2	1.12
IGFBP7	1.16
ISCU	-1.03
LARS2	-1.23
MRPS12	-1.78
MRPS17	-1.34
NDUFA12	-1.13
NDUFA4	-1.06
NDUFA5	-1.04

NDUFA7	-1.08
NDUFB8	-1.04
NEO1	1.14
PPOX	1.05
PRCP	1.08
SLC39A10	1.1
SNX21	1.16
STT3A	-1.13
TBRG4	1.07
VARS2	1.09
WARS2	1.17

Supplementary Table S7.

Supplementary Table S7A – Adipose: Expression of genes associated with disease and cellular functions, "inhibition of vasculogenesis" in adipose tissue of *Acss2*$^{-/-}$ compared to wild-type mice.

Symbol	Expr Log Ratio
ADIPOQ	1.08
CAV1	1
IL33	-1.24
JUN	-1.4
PPARG	-1.22
PPARGC1B	1.38
RHOB	-1.42
TKT	1.54
VLDLR	1.29

Supplementary Table S7B – Adipose: Expression of genes associated with disease and cellular functions, inhibited "infection of embryonic cell lines, infection of epithelial cell lines, and infection of kidney cell lines" in adipose tissue of *Acss2*$^{-/-}$ compared to wild-type mice.

Symbol	Expr Log Ratio
BMP1	-1.15
GANAB	-1.19
IRF3	-1.14
REPIN1	-1.2
RHOB	-1.42
RNPS1	-1.23
SF3A1	-1.25
TRAFD1	-1.2
ZYX	-1.38

Supplementary Table S7C – Adipose: Expression of genes associated with disease and cellular functions, "activation of glycolysis" in adipose tissue of *Acss2*$^{-/-}$ compared to wild-type mice. (**Note:** see also Figure 1 in main text).

**Symbol**	**Expr Log Ratio**
ADIPOQ	1.08
ALDOA	1.45
CAV1	1
ENO1	1.39
GAPDH	1.22
GPD1	1.7
GSTP1	1.18
IDH3A	1.19
MAPKAPK2	-1.22
MLXIPL	1.19
PDHA1	1.25
PFKL	1.4
PFKP	1.26
PPARG	-1.22
PPARGC1B	1.38
SLC2A3	-1.68
TPI1	1.19

Supplementary Table S8.

Supplementary Table S8A – Adipose: Expression of genes associated with **Network 1:** “cellular assembly and organization, RNA damage and repair, RNA post-transcriptional modification”, in adipose tissue of *Acss2*$^{-/-}$ compared to wild-type mice.

Symbol	Expr Log Ratio
ANLN	-1.47
BRD2	-1.24
CAMK2N1	-1.16
CDC42EP3	1.22
CDC5L	-1.29
CHD4	-1.21
CNIH4	1.13
CRYZL1	-1.23
DDX17	-1.67
DDX21	-1.21
DHX15	-1.16
DIDO1	-1.32
EEF1A1	-1
EFTUD2	-1.13
HDGF	1.07
HEXIM1	-1.32
KANK1	-1.26
MECR	1.24
MRPL12	1.2
NOL6	-1.29
PCBP1	-1.21

PIP4K2B	-1.22
PTPN6	-1.14
RNPS1	-1.23
RPL23	1.22
RSL1D1	-1.21
SLC25A10	1.78
SLC25A5	1.15
SLC4A1AP	-1.23
SRPK1	-1.24
SYF2	-1.23
THUMPD1	-1.27
TOP1	-1.12
UPF2	-1.33
ZCCHC8	-1.14

Supplementary Table S8B – Adipose: Expression of genes associated with **Network 2**: "connective tissue disorders, developmental disorder, hereditary disorder", in adipose tissue of *Acss2*$^{-/-}$ compared to wild-type mice.

**Symbol**	**Expr Log Ratio**
ACVR1C	1.6
ATP6AP2	-1.13
ATP6V0A2	1.21
ATP6V0E2	-1.26
ATP6V1A	1.41
BICD2	-1.23
BSDC1	-1.81

C1orf198	-1.3
CHORDC1	-1.31
CORO1A	-1.24
CORO1C	-1.31
FHL1	-1.28
GMFG	1.25
IFT172	-1.22
IFT20	1.12
IFT52	-1.33
IFT81	-1.15
JDP2	-1.12
LRRC41	-1.15
LUZP1	1.47
MYADM	-1.26
TMEM63B	1.39
USP16	-1.14
USP2	-1.79
USP22	-1.28
USP39	-1.25
WBP2	1.09
ZMAT3	-1.13
ZNRF2	-1.3
